# The Power of EI Competencies Over Intelligence and Individual Performance: A Task-Dependent Model

**DOI:** 10.3389/fpsyg.2018.01532

**Published:** 2018-09-07

**Authors:** Margarida Truninger, Xavier Fernández-i-Marín, Joan M. Batista-Foguet, Richard E. Boyatzis, Ricard Serlavós

**Affiliations:** ^1^ESADE Business School, Barcelona, Spain; ^2^Center for Creative Leadership, Greensboro, NC, United States; ^3^Geschwister-Scholl-Institute, Ludwig-Maximilians-Universität München, Munich, Germany; ^4^Department of Organizational Behavior, Case Western Reserve University, Cleveland, OH, United States

**Keywords:** emotional intelligence, emotional intelligence competencies, general cognitive ability, social tasks, analytic tasks, individual performance

## Abstract

Prior research on emotional intelligence (EI) has highlighted the use of incremental models that assume EI and general intelligence (or *g*) make independent contributions to performance. Questioning this assumption, we study EI's moderation power over the relationship between *g* and individual performance, by designing and testing a task-dependent interaction model. Reconciling divergent findings in previous studies, we propose that whenever social tasks are at stake, *g* has a greater effect on performance as EI increases. By contrast, in analytic tasks, a compensatory (or negative) interaction is expected, whereby at higher levels of EI, *g* contributes to performance at a lesser extent. Based on a behavioral approach to EI, using 360-degree assessments of EI competencies, our findings show that EI moderates the effect of *g* on the classroom performance of 864 MBA business executives. Whilst in analytic tasks *g* has a stronger effect on performance at lower levels of EI competencies, our data comes short to show a positive interaction of EI and *g* in affecting performance on social tasks. Contributions and implications to research and practice are discussed.

## Introduction

General intelligence, or *g*, has been the most studied and well-established predictor of professional and academic achievement. However, the last 25 years have witnessed the rise of emotional intelligence (EI), propelled by claims that it is superior to IQ in predicting performance (Goleman, 1995; Watkin, 2000). Defined as a set of abilities ranging from perception to regulation of emotions in the self and in others (Mayer and Salovey, 1997), the concept of EI has motivated extensive research aiming to show it has an incremental impact on performance (Lam and Kirby, 2002; Ferrando et al., 2011; Boyatzis et al., 2012). Yet, a recent source of controversy in the field concerns the increasing number of studies and meta-analyses attesting to mixed results (Van Rooy and Viswesvaran, 2004; Amelang and Steinmayr, 2006; Christiansen et al., 2010; Brackett et al., 2011). We contend that this lack of consistency across findings may be due, in part, to the predominance of models that measure the direct effect of EI on individual performance, above and beyond cognitive intelligence (known as incremental or additive models)—despite the fact that the scientific concept of emotional intelligence implies the integration of emotion and cognitive processes. Thus, paradoxically, multiplicative or interactive models have remained underexplored. Moreover, although it is clear that those who score high in both EI and *g* achieve top performances, little is known about predicting the performance of individuals with high levels of one ability and low of the other.

The present study thus examines the interaction effect of EI and *g* on individual performance. We do so in awareness that the first few studies of EI and *g*'s interaction on both job and academic performance are gathering further mixed findings. Notably, research conducted in organizations shows that EI and social skills improve the relationship between general mental ability and job performance. (Ferris et al., 2001; Verbeke et al., 2008; Kidwell et al., 2011). However, studies in academic institutions draw opposite results: researchers propose a compensatory model (or negative interaction) between EI and *g*, by which individuals' cognitive abilities have a smaller chance of contributing to performance whenever EI is high (Petrides et al., 2004; Côté and Miners, 2006; Agnoli et al., 2012; Fiori, 2015). In Casciaro and Lobo's (2005: 1) terms, these results seem to show that in academic settings, as opposed to organizational ones, it pays off to be a “competent jerk,” i.e., a person who's cognitively sharp but lacks the ability to communicate and relate with others.

In the attempt to reconcile such inconsistent findings between academic and organizational settings, we contend that whether EI and general mental ability interact in the way of complementing or compensating each other ultimately depends on the type of task being performed. As such, adopting Jack et al.'s (2012) categorization of tasks, we hypothesize that in *social* tasks, EI and *g* may function as complements, mutually reinforcing their effects on performance. By contrast, in *analytic* or non-social tasks, when EI is high, we may observe that performance is less vulnerable to the level of cognitive ability, since individuals are able to use their EI competencies as a coping device whenever they lack the intellectual ability to perform the task successfully. As such, we design a task-dependent interaction model of EI and *g* on performance, and test it on a population of managers and business executives enrolled as part time candidates in an international MBA program at a leading European business school.

Overall, this study offers three valuable contributions to the EI-performance research: First, because “worthwhile findings may emerge from research utilizing other approaches to assessing EI [other than Ability EI or Trait EI], including multisource (‘360°') assessments, videotapes of simulations or behavioral coding of taped interviews (Boyatzis, 2009; Boyatzis et al., 2012).” (Webb et al., 2013: 155), our study assesses individuals' behavioral manifestations of EI as rated by external informants from the professional context (i.e., bosses, peers and direct reports). Second, it unveils the understudied moderator role of EI on the relationship between *g* and performance. Third, it internalizes task-dependence in the analysis, by considering two types of tasks (social and analytic) within the same sample. In so doing, we are first, to our knowledge, to examine the interactive effect of a behavioral measure of EI on individual performance.

## Behavioral emotional intelligence

A scientific conception of emotional intelligence first came to form in 1990 as a true intellectual ability that met traditional standards for an intelligence (Salovey and Mayer, 1990; Mayer et al., 1999). EI has since been defined as a set of interrelated skills, including “the ability to perceive accurately, appraise, and express emotion; the ability to access and/or generate feelings when they facilitate thought; the ability to understand emotions and emotional knowledge; and the ability to regulate emotions to promote emotional and intellectual growth.” (Mayer and Salovey, 1997: 10).

Despite EI's field being deep in controversy with several definitions and assessments over its first 25 years of research, emotional intelligence, as a concept that comprises a set of inter-related abilities pertaining to the perception and regulation of emotions in the self and in others, provides a common content domain to existing EI measures (Joseph et al., 2014). What essentially distinguishes existing EI models is their choice of measurement theory, a decision that is grounded in the level at which one chooses to observe EI: be it as a mental ability, a trait of personality, or an actual behavior as assessed by others.

In perhaps the most well-established classification of EI research (Ashkanasy and Daus, 2005) contend there are three streams or approaches to EI: While Streams 1 and 2 are solely devoted to measures based on Mayer and Salovey's (1997) Ability EI model–assessed either by performance tests (MSCEIT; Mayer et al., 2003) in Stream 1 or peer-reports and self-assessments in Stream 2 (e.g., Schutte et al., 1998; Jordan et al., 2002; Wong and Law, 2002)—all other remaining EI models have been clustered in Stream 3, or what has been labeled the “mixed EI” approaches (Mayer et al., 1999). Referring to the obscure nature of this label, Joseph et al. (2014: 2) called it a “black box” and noticed how prior theoretical work on mixed EI is scant. To be sure, mixed EI has never been conceptualized. In fact, it is seldom used in any of the EI approaches it represents. Rather it is a “uninformative label” created to designate all research on EI that does not use or adapt Ability EI's model (Boyatzis et al., 2015). Consequently, recent research (Cherniss and Boyatzis, 2013; Amdurer et al., 2014; Boyatzis et al., 2017) proposes that Stream 3 be split further to allow distinguishing Trait EI (Stream 3), which assesses EI through self-reports of personality traits, attitudes and motivations (e.g., EQ-i; Bar-On, 1997a), from Behavioral EI (Stream 4), an approach that captures EI as is manifested in real contexts by collecting external informants' observations (as opposed to self-assessments) of an individual's behavior (ESCI; Boyatzis and Goleman, 2007). This distinction enables future meta-analyses to properly differentiate all existing EI approaches. After all, “[all] these approaches try to discover *the emotional components that underlie emotionally intelligent people* and the mechanisms and processes that set off the use of these abilities in our everyday life” (Fernández-Berrocal and Extremera, 2006: 8, emphasis added).

In this paper we use the behavioral approach to EI, for it allows capturing emotional intelligence at a level that is closer to action and consequential to real-life and work performance, i.e., actual behavior in situated contexts. Considering that the etymological roots of emotion come from the Latin word *emovere*, a combination of *ex* (out) + *movere* (to move) is a good reminder that emotion is strongly associated with external movement that provides signals to others. Darwin's (1872) treatise on emotional expression performed a comparative study of humans and animals and gathered unequivocal evidence on the breadth of emotional communication that is captured through body movements and facial expressions. Similarly, emotional intelligence can be seized in both verbal and non-verbal behavior that is visible and consequential to others, offering a sound basis to establish a behavioral approach to EI.

Conceptually, behavioral EI concerns the same content domain as other EI approaches, i.e., the concept of emotional intelligence as defined in Salovey and Mayer (1990). Specifically, the Emotional and Social Competency Inventory model (ESCI; Boyatzis, 2009) parallels the original definition of EI, in that it contains behavioral indicators of competencies that reflect: (1) the same core abilities of awareness (or perception) and management (or regulation) of emotion; and (2) the same targets, that is, whether these abilities are directed at self or others. In a critical review of the field, Zeidner et al. (2004) clarifies that what differentiates the approach of ability EI from its behavioral counterpart is akin to the distinction between fluid and crystallized ability. As the authors explain: “EI (as a fluid ability) does not guarantee that individuals will actually manifest competent behaviors at the workplace. (…) Whereas [ability] EI may determine a person's potential for learning practical job-related emotional and social skills, the level of emotional competencies (as a crystallized ability) manifested by that person shows how much of that potential she or he has actually realized” (Zeidner et al., 2004: 377). Indeed, some individuals may be good at mindfully thinking and coming up with solutions to hypothetical emotional-laden problems, but lack the training or experience for actually performing the behaviors they prescribe (Fiori, 2009).

The ESCI model used in this paper is empirically supported by 40 years of research identifying competencies that predict work success (McClelland, 1973; Boyatzis, 1982; Spencer and Spencer, 1993). Competencies have been defined as learned capabilities that lead to effective or superior performance and are reflected by a set of behaviors that share a common underlying intent (Boyatzis, 2009). Because the identification of competencies and their refinement emerges from a performance based criterion sampling, they are expected and in fact have been shown to consistently predict academic, job and life outcomes (Boyatzis, 1982, 2006; Spencer and Spencer, 1993; McClelland, 1998; Dulewicz et al., 2003; Law et al., 2004; Boyatzis et al., 2011, 2012; Amdurer et al., 2014; Mahon et al., 2014).

## EI, cognitive ability and performance

Throughout the past century, general mental ability, also known as general intelligence, general cognitive ability or simply *g*, has taken the leading role in enlightening our understanding of human performance (Fiori and Antonakis, 2012; Nisbett et al., 2012; Webb et al., 2014). As a global ability concerning the “general efficacy of intellectual processes” Ackrman et al., (2005: 32), *g* is thought of as the apex or common factor to all types of specific intelligences—e.g., fluid intelligence (Gf), crystallized intelligence (Gc), broad visual perception (Gv), broad auditory perception (Gu), broad cognitive speediness (Gs), etc. (Carroll, 1993; McGrew, 1997). As a latent construct, *g* is not observed directly; rather, it is inferred from the positive correlations among specific mental abilities (Spearman, 1904; Jensen, 1998). A large body of evidence shows *g* has a strong relationship to school and workplace performance across tasks and settings (Gottfredson, 1997; Jensen, 1998; Ree and Carretta, 1998; Schmidt and Hunter, 1998; Ree and Carreta, 2002; Salgado et al., 2003).

But, although *g* correlates between 0.30 and 0.50 with several performance measures, it actually only explains about 25% of their variance (Hunter and Hunter, 1984; Goldstein et al., 2002). For this reason, the case for EI has been built over claims that it explains variance in performance that has not yet been accounted for by cognitive intelligence (Goleman, 1995; Mayer and Salovey, 1997; Mayer et al., 2000; Watkin, 2000). This argument has led researchers to put an emphasis on identifying direct additive effects of emotional intelligence on performance, which assume emotional intelligence and cognitive intelligence make independent contributions to human performance. Paradoxically though, this assumption of independence is in contradiction with the very concept of emotional intelligence, by which “emotion makes thinking more intelligent and that one thinks intelligently about emotions.” (Mayer and Salovey, 1997: 5). Furthermore, the original conceptualization of EI emerged from an important neuroscience discovery: the integration of emotion within cognitive processes across a variety of mental functions such as memory, attention, and decision-making (Mayer and Bremer, 1985; Forgas and Moylan, 1987; Damasio, 1994). Hence, additive models may in fact be too “simplistic and incomplete” to represent the contribution of EI to performance (Côté and Miners, 2006: 2).

In what regards the relationship between EI and workplace performance, recent meta-analyses suggest that emotional intelligence positively affects several aspects of workplace performance (Joseph and Newman, 2010; O'Boyle et al., 2011), including company rank and pay increases (Lopes et al., 2006) and supervisor ratings (Côté and Miners, 2006). In particular, emotional and social competencies have been shown to positively affect sales leadership performance (Boyatzis et al., 2012), management (Ramo et al., 2009; Boyatzis et al., 2012), entrepreneurship performance (Camuffo et al., 2012) and engineers' effectiveness and engagement (Boyatzis et al., 2017). Other studies, however, find significant relationships between EI and workplace performance but do not take general mental ability into account (Bar-On, 2000; Law et al., 2004).

As to EI's effect on academic performance, previous studies report conflicting findings (Brackett et al., 2011): whereas some research shows that EI explains achievement in high school (Gil-Olarte Márquez et al., 2006) and undergraduate programs (Lam and Kirby, 2002), other studies suggest there is no relation or a non-significant one between emotional intelligence and academic performance (Petrides et al., 2004). In fact, it is often the case that studies will initially show positive effects of EI on performance until they eventually become non-significant after controlling for variables such as cognitive intelligence and personality traits (Barchard, 2003; Brackett and Mayer, 2003). Such large variation across studies is leading EI researchers to adopt multiplicative models, whereby the interaction effect of EI and *g* on performance is explored (e.g., Petrides et al., 2004; Côté and Miners, 2006; Kidwell et al., 2011).

Moreover, prior research has devoted little attention to examine how EI may relate differently to performance depending on the *type* of task (Rode et al., 2007). Notably, EI may be especially relevant in tasks that require social interactions (Lopes et al., 2004) and group processes (Druskat and Wolff, 2001; Jordan and Troth, 2004). For this matter, we propose a task-dependent model of the interaction between EI and *g* on individual performance.

## A task-dependent interaction model of EI, *g* and performance

We propose that the interaction between EI and *g* on human performance depends on the type of task. We use a taxonomy of tasks that takes into account the *content* of the information processed (Jack et al., 2012; Friedman et al., 2015). According to Jack et al. (2012) task content is based on two opposing cognitive domains: The *social* cognitive domain relates to tasks that require social information processing, i.e., reasoning about the minds of others and/or involving interpersonal interaction, which plays a leading role in emotional self-awareness, social cognition and ethical decision making. The *analytic* (or non-social) cognitive domain pertains to tasks that require reasoning about the causal or mechanical properties of inanimate objects, thought to be most relevant for problem solving, focusing of attention, making decisions and action control (for a review see Boyatzis et al., 2014).

The study of these two types of tasks within a single sample requires choosing a setting that enables the separation between social and analytic tasks. One advantage of conducting this study in an academic setting stems from the ability to distinguish social from analytic tasks within performance units, seen that we can select distinct courses with significantly different amounts of social and analytic tasks. For example, courses such as Marketing or Leadership that involve reflecting about human decision-making and social information processing can be said to include substantially more social tasks (i.e., discussions about human behavior and interpersonal interaction), than analytic courses such as Finance or Statistics, which revolve around abstract and arithmetic concepts. Specifically this study is conducted within an international MBA program, wherein the classroom performance of business executives in both social and analytic courses is assessed.^1^

### Social tasks

When individuals engage in social tasks, what is the interactive nature of the relationship between EI and *g* on performance? We propose that when individuals have low levels of EI competencies, such that they can't get along with others, they will have poor performances, regardless of their level of intelligence. Their inability to relate with and understand others hinders their capacity to collaborate and be of service to others, which ultimately compromises their overall performance. An anecdote typically found in organizations illustrates this: “She may be a genius, but she isn't getting things done here because she can't work with people.” In Casciaro and Lobo (2005)'s terms this would correspond to a “competent jerk,” who regardless of how intelligent he may be, he will still end up being dismissed by others, which in non-modular work that necessarily involves interpersonal interactions, it will lead to a poor performance. This way, at low levels of EI the relationship between *g* and performance is significantly attenuated.

Conversely, high levels of emotional intelligence act as a catalyzer or booster of the relationship between cognitive intelligence and performance. This way, even extremely intelligence people can further heighten their performance by learning EI competencies that enable them to share information and effectively collaborate with others. Notably, referring to the myth of the lone genius that pervades our society, Shenk (2014) exposes an unexpected finding: the lone geniuses are just the most well-known halves of collaborative duos. Interestingly, examples abound: Lennon-McCartney, Newton-Halley, Einstein-Besso or more recently Kahneman-Tversky. Notably, the Economics Nobel Prize winner, Daniel Kahneman, illustrates his collaboration with Amos Tversky, as follows: “*Indeed, one of the great joys in the collaboration was that Amos frequently saw the point of my vague ideas much more clearly than I did. For the next fourteen years our collaboration was the focus of our lives, and the work we did together during those years was the best either of us ever did*.” Kahneman (2011: 5–6). Thus, in social tasks, EI and *g* contributions to performance are mutually reinforcing such that they act as *strategic complements*.^2^ Therefore, we propose the following:

*Hypothesis 1. In social tasks, the relationship between general mental ability (or g) and performance is stronger among individuals at higher levels of EI competencies*.

### Analytic (or non-social) tasks

When engaging in analytic tasks, which require practically no social skills, we expect that the performance of individuals at lower levels of EI competencies will be more vulnerable or sensitive to their level of cognitive abilities. While individuals with low EI and low *g* will have bottom low performances, those with low EI but high *g*, will be able to significantly compensate for their shortage of EI competencies by investing on or deploying their cognitive abilities. This is particularly so because cognitive skills may not only be necessary but sufficient to accomplish analytic tasks successfully.

Conversely, at higher levels of EI competencies, performance becomes less vulnerable to an individual's level of cognitive abilities. On one hand, individuals with high EI but low *g*, can effectively deal with feelings of frustration and withdrawal whenever facing cognitively challenging tasks. By deploying EI competencies such as emotional self-awareness, emotional self-control, achievement orientation or positive outlook, they are better able to cope with and manage emotions in such a way that motivates and energizes them to be confident in their ability to learn and to adopt a growth mindset, setting them up in a path to greater performance. This way, once an individual has high EI competencies—particularly those related to self-awareness and self-management—moving from low to high *g* has a smaller impact on performance (as compared to having low EI), since these individuals are well-equipped to cope with and overcome the emotional turmoil of facing cognitively challenging tasks, keep calm and move on to reach higher levels of performance.

As such, in analytic tasks, EI and *g* have a compensatory effect (or negative interaction) on performance. In result, these two abilities may be perceived as *strategic substitutes*,^3^ whereby individuals might have a greater incentive to invest in learning EI competencies, the lower their cognitive skills are. From the preceding discussion we suggest the following hypothesis:

*Hypothesis 2. In analytic tasks, the relationship between general mental ability (or g) and performance is weaker among individuals at higher levels EI competencies*.

**Figure 1:**
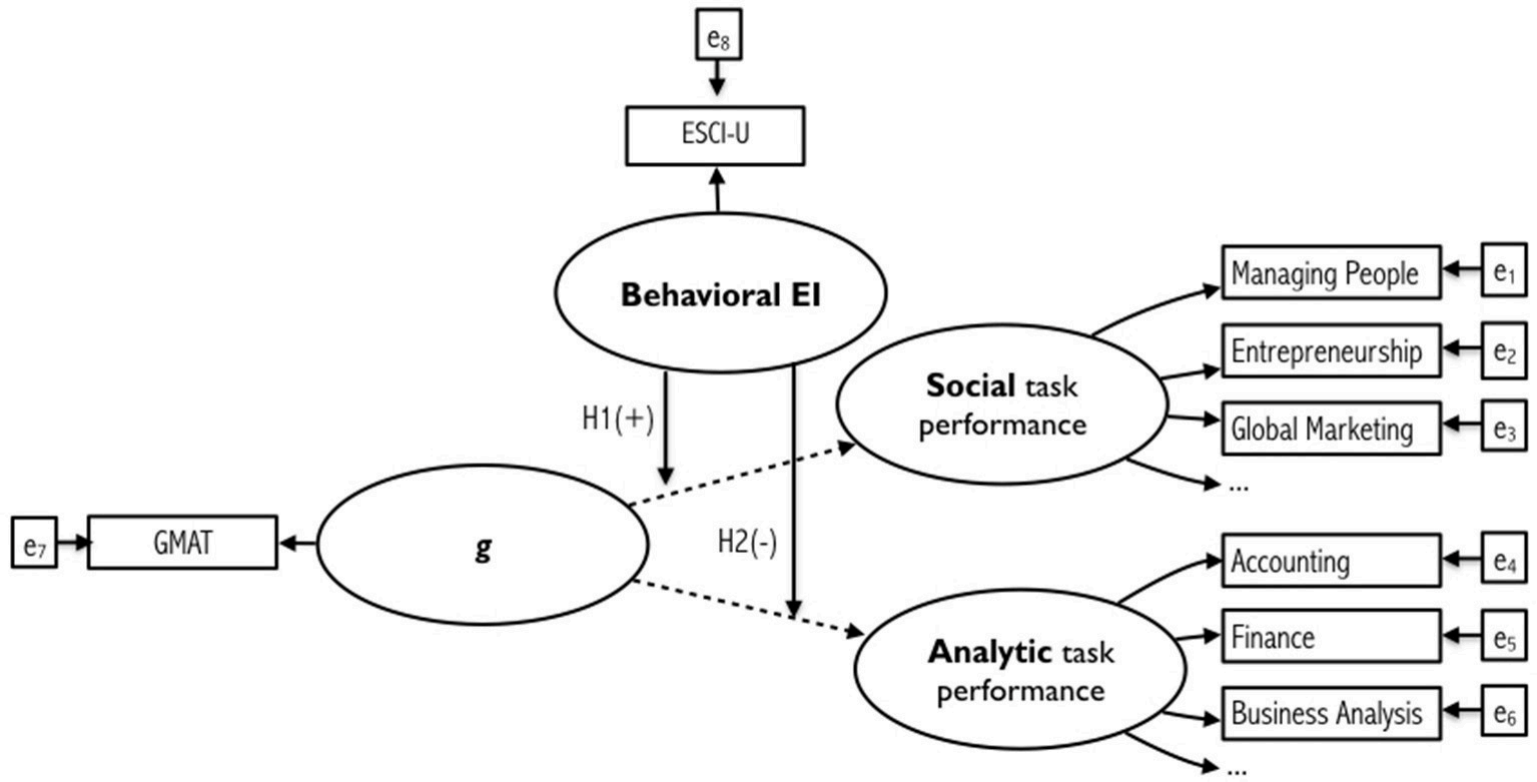
Path diagram of the task-dependent interaction model of EI and general intelligence on the individual performance on social vs. analytic tasks. Dashed lines represent direct effects that have been previously tested in earlier research and thus are not represented in our hypotheses.

Figure 1 below shows the overall path diagram of the task-dependent interaction model of EI competencies and cognitive ability for enhancing performance, including both structural and measurement relationships.

## Data and methods

### Participants

Data were collected on 864 managers and business executives enrolled in part-time and executive MBA programs at a top European business school, between 2006 and 2013. There were 31% females, and the average age was 29 years (SD = 2.6). As part of the MBA, the candidates took the compulsory course of Leadership Assessment and Development, which is based on the Intentional Change Theory (Boyatzis, 2008), and is specifically designed to develop emotional and social competencies. In this course, the candidates completed a self-assessment and selected multiple raters from their professional sphere (i.e., bosses, peers and direct reports) to provide a 360 appraisal of their EI competencies. All data were collected under the informed consent and ethical guidelines of ESADE Business School.

### Measures

#### Behavioral EI

We used the Emotional and Social Competency Inventory-University Edition (ESCI-U; Boyatzis and Goleman, 2007). The ESCI-U comprises 12 EI competencies organized into four clusters corresponding to the Cartesian product between EI abilities (awareness/management of emotion) and the target: self/others. Those clusters are: *Self-awareness*, which includes the competency of emotional self-awareness; *Self-management*, including emotional self-control, adaptability, achievement orientation and positive outlook; *Social awareness*, which consists of empathy and organizational awareness; and *Relationship management* comprising coach and mentor, inspirational leadership, influence, conflict management and teamwork. Each EI competency is measured with five behavioral indicators (totaling 60 indicators).

The ESCI model measures behavioral EI as is seen and assessed by others. For this matter, it uses a 360° assessment instrument (Sala, 2002; Boyatzis and Sala, 2004; Boyatzis and Goleman, 2007), which enables multiple raters from the professional sphere—namely bosses, peers and direct reports, to provide behavioral observation scores of the person being assessed. The ESCI-U has provided evidence of construct and discriminant validity (Byrne et al., 2007; Cherniss, 2010; Cherniss and Boyatzis, 2013; Boyatzis et al., 2015). The ESCI-U asks both self-report and external raters to score the frequency of each behavioral indicator on an 11 point-scale going from 0, “the behavior is never shown” to 10, “the behavior is consistently shown.” The 11-point scale has been shown to be superior to the 5-point scale for rating frequency (Batista-Foguet et al., 2009).

#### General mental ability (g)

We used the Graduate Management Admission Test (GMAT) to measure general mental ability or *g*. The GMAT is a standardized test for admission into graduate management programs. It assesses analytical, mathematical, writing and reading skills, across which the underlying common factor is interpreted as the *g* factor. Several studies before ours have used GMAT as a measure of *g* (e.g.,O'Reilly and Chatman, 1994; Kumari and Corr, 1996; Hedlund et al., 2006; Mueller and Curhan, 2006; Boyatzis et al., 2015), including a study published in *Intelligence* (Piffer et al., 2014). For instance, Hedlund et al. (2006: 102) concludes that “like the SAT [a standardized test which has been shown to be a valid measure of *g* (Frey and Detterman, 2004)], the GMAT can be characterized as a traditional measure of intelligence, or a test of general cognitive ability (*g*).”

#### Individual performance

We measured performance by computing scores for the MBA candidates' grading performance at social and analytic courses, based on a total of 45 course gradings collected at the university registrar after the end of each term. In order to classify the courses as social or analytic, we created a set of 16 binary indicators to code each course syllabus for the presence of social content (10 words pertaining to social content were used, such as “human” or “society”) and social interactions (i.e., degree of teamwork, use of debates in class). Two raters coded the 45 courses' syllabi and achieved an inter-rater reliability of 88%, well above the generally accepted threshold of 70% (Stemler, 2004). Next, we used an item-response theory model to estimate a single latent dimension, upon which only the courses with loadings higher than 1 in absolute value were kept for the analysis. Therefore, courses such as Entrepreneurship, Managing People or Global Marketing, with loadings higher than 1 were labeled “Social,” while Accounting I, Finance or Business Analysis to Valuation, with loadings below −1, were labeled “Analytic.” The performance scores for each type of course were then obtained by computing simple averages across individual grades. Each individual grade was however a standardized score of the position of the individual in the group/year for a given course. Standardizing by group/year is an efficient way of eliminating professor effects, or the differences in ratings regarding the idiosyncrasies of certain topics. So what we are really measuring is how well students perform as compared to other students (or, in other words, considering the group), rather than raw grade performance.

### Procedures

In agreement with Anderson and Gerbing (1988), the data analysis process is divided into two different models: the measurement model, aforementioned, and the explanatory or structural model.

The explanatory model is a non-nested hierarchical robust linear model between individual performance and the covariates (GMAT, ESCI, the interaction between the two, and gender as a control variable—measured with a dummy variable that takes the value 1 if gender is Female). The hierarchical structure is necessary in order to account for the different ways in which the data is naturally structured: first because there are two measures of performance per individual (social and analytic course performance) and some of the effects may or may not be shared across the two types of courses; and second, because emotional and social competencies are measured in a two-level cluster: competencies and competency clusters. Equation (1) below describes the explanatory model of the linear association between performance (for two different types of task *t*) and the covariates (*X*), when ESCI competencies (*c*) are measured by professional raters (i.e., bosses, peers and direct reports), and organized in clusters (*cl*) and higher-level clusters (*CL*), for each of the individuals (*i*).

(1) $$\begin{array}{r} \begin{array}{c} Y_{i}\sim T\left(\mu_{i},\sigma_{i},\upsilon \right)\\ \mu_{i}=\alpha_{t,c}+\left(Female_{i},GMAT_{i},ESCI_{i},GMAT_{i}*ESCI_{i}\right)\theta_{t,c}\\ \theta_{t,c}\sim N\left(\Theta_{t,cl},\sigma_{\theta_{t}}\right)\\ \Theta_{t,cl}\sim N\left(\mu_{\Theta_{t,CL}},\sigma_{\Theta_{t}}\right)\\ \mu_{\Theta_{t,CL}}\sim N\left(0,100\right)\\ \sigma_{i}=\exp\left(Intercept_{i},Female_{i},GMAT_{i},ESCI_{i,c}\right)\lambda \\ \lambda \sim N\left(0,10\right)\\ \upsilon \sim U\left(1,20\right) \end{array} \end{array}$$

Equation (1) can be read as follows: Performance for any individual in any of the two types of tasks (social or analytic) is a linear combination of an intercept (α), the direct effect of general intelligence, the direct effect of EI and, lastly, the interaction effect between general intelligence and EI, controlling for gender.

Inference is performed using Bayesian procedures, namely the Gibbs sampler and MCMC methods using the ggmcmc R package (Fernández-i-Marín, 2016). There is one good reason to prefer Bayesian inference for addressing our research: Our data was not drawn from a random sample. This would pose a problem if we were to use traditional frequentist methods since these are based upon the assumption that the data are created by a repeatable stochastic mechanism. While frequentist statistics treats the observable data as random and the unknown parameters of the population as fixed and unchanging, in the Bayesian view, it is the observed variables that are taken as fixed (which in reality, that is what happens, at least in the social sciences: seldom do researchers collect 100 samples), whereas the unknown population parameters are assumed to vary randomly according to a probability distribution^4^.

Overall, the main advantages of the Bayesian approach are 2-fold: (1) it is more appropriate for settings where the data is not a random sample; and (2) it enables highly flexible model specifications (as the one needed to account for the hierarchical structure of our data). Furthermore, it offers a clear and intuitive way to present results, by generating probability statements about the findings (for more readings on the advantages of Bayesian inference, see the introductory chapters of Gill, 2002; Gelman et al., 2003; Jackman, 2009).

## Results

**Figure 2:**
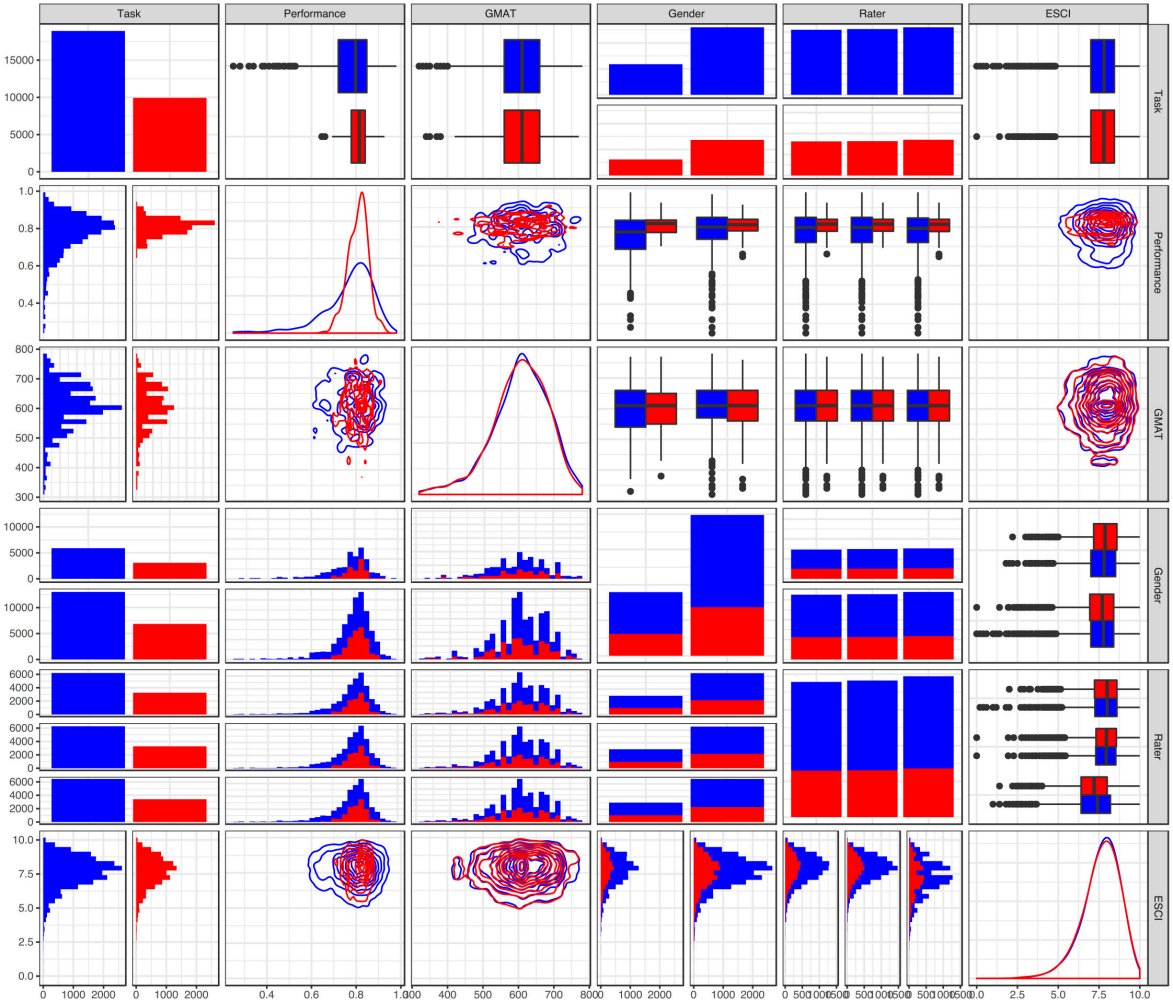
Diagonal figures represent the univariate distribution of each of the variables (densities for continuous variables, bar plots for binary variables). Upper and lower triangle figures are bivariate distributions of the values. In the case of two continuous variables, instead of a dotplot we have used a bivariate density plot. The bivariate density plot represents a view from the top, with the lines highlighting areas with increasing density, as in a topographic map. In all cases, colors represent social (red) and analytic (blue) tasks.

Figure 2 below offers a visual summary of the descriptive statistics of the individuals, regarding the key variables in our model.

Regarding the ESCI, Figure 2 shows that its density distribution has a negative skew, with higher probabilities associated with upper values, such that ESCI's overall mean is 7.64 (SD = 1.16). In what concerns the 360° evaluations, there were, on average, 4.2 (SD = 1.5) raters per MBA candidate who provided feedback on their emotional and social competencies. To summarize the 360° assessments on the ESCI questionnaire, we first obtained for each behavioral indicator, the average score across the professional raters. Then we averaged across the five items per each competency to finally obtain one score per competency. Concerning the GMAT, its sample mean of 602.6 was a little higher than the overall population mean of 545. GMAT's sample standard deviation of 78.8 was approximately two thirds of its population counterpart at 121. The figure above shows a slight negative skew, with considerably higher values for males than females. As to performance, while in analytic courses (in blue) the distribution is negatively skewed, in social courses (in red) it is approximately normal.

**Figure 3:**
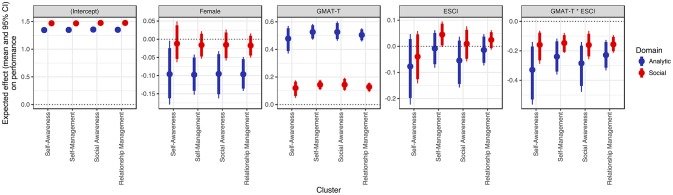
Coefficient estimates of the direct effects of ESCI, as assessed by professional raters, GMAT and the interaction effect of ESCI*GMAT on individual performance.

Figure 3 shows the coefficient estimates, obtained through robust regression, of GMAT, ESCI, and the interaction effect between ESCI and GMAT on the performance in social and analytic types of courses. The ESCI estimates are presented in terms of the four clusters: *Self-awareness, Self-management, Social awareness*, and *Relationship management*. Analogous to a measure of effect size, the Bayesian approach to regression modeling produces estimates of the percentage decrease in the residuals' standard deviation, an approximate measure of (1 – *R*^2^). As such, the regression on social tasks resulted on an average decrease in the residual standard deviation of 2.34%, whereas the one on analytic tasks produced a decrease of 8.88%. Reading the panels in Figure 3, there are three main findings: (1) GMAT has a positive effect of 0.5 grade points on the performance in analytic courses, which is significantly higher than the 0.1 grade points in social courses; (2) The direct effect of ESCI on performance is inconclusive, with some of the clusters such as *Self-management* having a negative effect on performance, while others having no effect or a slightly positive one, thus, confirming the mixed findings in previous EI-performance studies using additive models; and (3) There is a significant interaction effect of ESCI and GMAT on performance and it is negative for both types of tasks. This finding informs the central research question in this study. While it supports hypothesis 2 of a negative interaction between EI competencies and general intelligence on the performance of analytic tasks, it shows no support for hypothesis 1. However, there is a positive second order interaction, by which the interaction effect increases from analytic to social courses, i.e., social tasks have a closer to positive interaction than analytic tasks do. This finding may be viewed as offering a hint of support to hypothesis 1.

**Figure 4:**
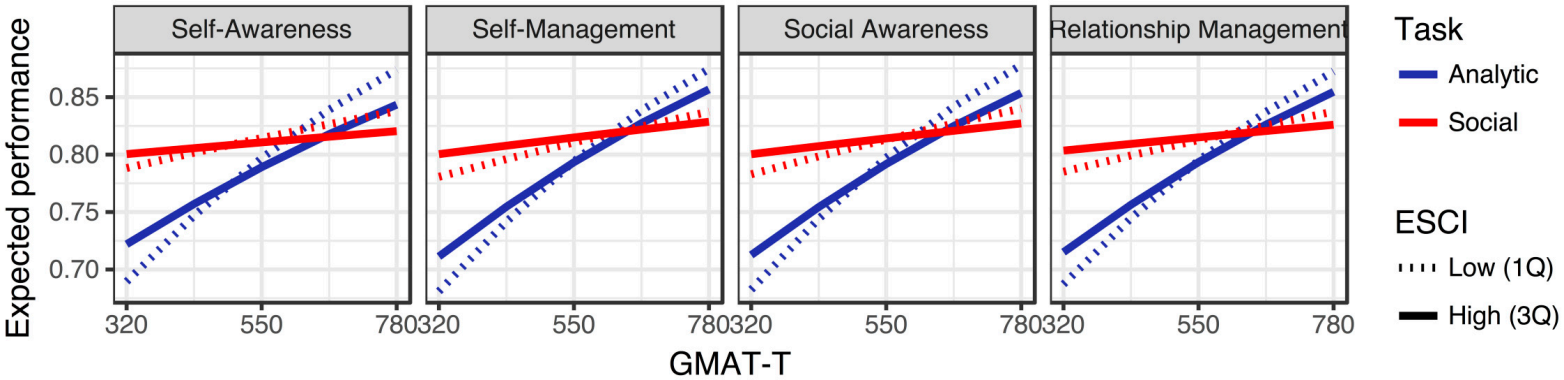
Interaction effect between ESCI and GMAT on the individual performance on social and analytic tasks.

Figure 4 provides a visual summary of the moderating effect of ESCI on the relationship between GMAT and individual performance in social and analytic courses. Expected performance is shown in red for social courses and in blue for analytic courses. Dashed lines represent individuals scoring below the 25th quantile of ESCI scores, whereas solid lines represent the top 25% individuals with the highest ESCI observed. The horizontal axis accounts for the range of potentially observed GMAT values. As expected the effect of GMAT on performance is positive in both types of tasks with a higher effect on analytic than social courses. As ESCI shifts from low to high levels the effect of GMAT on performance decreases (the slope becomes flatter). However, this decrease is lower in social than analytic tasks, which, as aforementioned, offers a hint of support to hypothesis 1.

## Discussion

Earlier research has proposed that emotional intelligence and cognitive abilities contribute to performance in independent ways (Mayer et al., 2000). The present study shows that rather than an incremental additive effect on performance, EI, as measured by a behavioral assessment instrument, has a multiplicative effect: Specifically, behavioral EI moderates the relationship between *g* and the individual performance of business executives. As predicted in hypothesis 2, we find evidence that in analytic tasks, the higher individuals' EI competencies the weaker is the effect of their cognitive skills on performance, or in other words, the less vulnerable is performance to their levels of cognitive intelligence. In agreement with Côté and Miners (2006), Agnoli et al. (2012), and Petrides et al. (2004), we find that those individuals with high EI competencies but low cognitive abilities, whenever faced with cognitively challenging tasks, are able to compensate for their performance by deploying EI competencies. Facing a cognitive challenge (i.e., when a tasks' intellectual demands outweigh one's cognitive abilities), can be emotionally taxing. As sentiments of fear and frustration emerge, they may sabotage one's focus and approach motivation. In these situations, having trained and developed EI competencies, especially those related to self-awareness and self-management of emotion—such as emotional self-awareness, emotional self-control, adaptability, achievement orientation or positive outlook—enables individuals to take distance from their distressing emotions and re-appraise the cognitive challenge as an opportunity to learn rather than a threat, enabling them to keep calm, confident and focused throughout the task. As such, in analytic tasks, cognitive intelligence has a weaker impact on performance at higher levels of EI competencies.

In practice there is, however, a moral downside to a compensatory (or negative) interaction between EI and *g* on performance: it suggests that emotional intelligence is not as beneficial to those individuals at higher levels of cognitive intelligence. In fact, there is a widespread shared understanding that highly intelligent individuals do not really need to learn emotional intelligence skills, since their cognitive abilities are high enough to get them all the success they want. Naturally, this prediction may only make sense in tasks that do not require collaboration or inter-personal interaction whatsoever.

Conversely, when tasks engage the social cognitive domain and require social understanding or interpersonal interaction to be accomplished, high cognitive skills alone may not be sufficient to succeed. Hypothesis 1 proposes that in social tasks, cognitive intelligence becomes more consequential to performance when coupled with stronger rather than weaker emotional intelligence competencies. i.e., a positive interaction, wherein EI and *g* mutually reinforce each other's contributions to performance. Although our data did show a positive second order interaction, by which there was a relatively higher interaction between EI and *g* on the performance of social as compared to analytic courses, this increase was not sufficient to support hypothesis 1.

We suspect hypothesis 1 found little support in our data due perhaps to a lack of distinction between social and analytic tasks across our business school's MBA courses. Although it is reasonable to consider that social courses, such as Marketing or Management, offer humanistic and social related content, providing greater opportunities for debate, teamwork and, thus, interpersonal interaction, this may not always be the case in practice. Teamwork takes time to be fruitful, and often leads to conflict and wasted time before real collaboration takes place. Perhaps because our MBA programs are organized into short trimester courses (rather than semesters), students may choose to work by themselves rather than lose time trying to figure out how to deal with cross-cultural teams in such short time period. Indeed, early on into their MBA program candidates might find that splitting up teamwork projects into individual components is more efficient than engaging into actual teamwork, regardless of course content. Therefore, even if social courses should normally require more discussion and interaction within teams, the fact that students forge an individual work system in most courses, wherein they experience minimal to no personal interaction among team members, may blur the distinction between social and analytic tasks.

### Limitations

A first limitation in this study concerns the range restriction in the GMAT, our measure of general cognitive ability. This is due to an MBA admission criterion that requires candidates to score above a certain threshold in their GMAT (usually above 600 points). Our attempt to correct for range restriction, by using the students' GMAT scores collected from the first time they took the test, as opposed to the scores with which they were admitted in the MBA (scores that may have been obtained after attempting the test several times), was effective insofar as it increased the variation in GMATs, but was limited to solve the selection bias within our sample. As such, our ability to make inferences at lower levels of GMAT was compromised.

Moreover, our performance measures were based on grades from various MBA courses. Teachers' assessments of performance may be biased by the quality of relationships they establish with students, a phenomenon known as leader-member exchange (Graen and Uhl-Bien, 1995), which we were unable to control for.

Finally, the fact that our data was collected in one specific school may threaten the external validity of our findings.

### Main contributions and future research

To our knowledge, so far only six studies have examined the interaction between EI and cognitive ability on academic and job performance; although all have found statistically significant interactions, some were positive (in job settings) and others negative (in academic settings; Petrides et al., 2004; Côté and Miners, 2006; Verbeke et al., 2008; Kidwell et al., 2011; Agnoli et al., 2012; Fiori, 2015). We join their shared call for further research that moves beyond incremental effects and pays attention to the interaction of EI with interdependent intelligences, such as cognitive ability. This involves recognizing the false myth in our scholarship by which EI, or any other construct for that matter, may only be valuable for research and practice, if it makes an incremental and independent contribution to performance (Zeidner et al., 2004; Landy, 2005). Rather, emotional intelligence, as a predictor of human performance, can be particularly more important and consequential in multiplicative ways (Murphy, 1996; Hough, 2003). Exploring these interactive paths enables researchers to discover EI is valuable because it moderates or determines the extent with which other variables affect performance.

The main contribution this paper offers to future research lies in the theoretical framework we develop for studying the interaction of EI and *g* on performance: the task-dependent interaction model of EI. By internalizing distinct types of tasks, social and analytic, within the same sample, this model provides a potential way to reconcile the divergent findings among previous interaction studies conducted in organizational and academic settings. In agreement with Rode et al. (2007), EI may be significantly more helpful whenever tasks require a high degree of interpersonal interaction, an observation that has been thoroughly explored in preliminary research studying the impact of EI on group processes (Druskat and Wolff, 2001, 2008; Jordan and Troth, 2004) and the quality of social interactions (Lopes et al., 2004). Therefore, we encourage researchers to explore task-dependent models, such as the one found here, for considering multiplicative effects of EI on human performance.

However, insofar as academic tasks are modular (i.e., that can be broken down into smaller and independent tasks), and do not require students to engage in de facto collaboration, we may be at odds to observe the catalyzing power (i.e., positive interaction) of EI competencies on the relationship between cognitive ability and performance in academic contexts. As such, the replication of this study in organizations—where a majority of work is done in teams and require interpersonal interaction (Druskat and Wolff, 2001)—could offer a better chance to gather evidence in support of hypothesis 1 in our model, wherein emotional intelligence enhances the effect of intelligence on performance, whenever social tasks are at stake. Evidence in support of this hypothesis might provide the much needed incentive for “competent jerks” (Casciaro and Lobo: 1) to learn EI competencies so as to effectively collaborate with others.

In addition, our results, together with previous work (Furnham et al., 2014; Boyatzis et al., 2015), show the importance of considering 360-degree behavioral assessments of EI. People at different organizational levels may have unique vantage points from which to observe distinct facets of behavior, depending on the specific relationship and rapport they have with the person being assessed. Therefore, we suggest future research should benefit from introducing multisource assessments within different EI measures. Specifically, it would be interesting to explore the distinctive perspectives across the various sources within professional but also personal contexts (such as friends, relatives, partners, etc.), and look into identifying which particular competencies each rater is best able to observe and assess.

Finally, we join researchers working on different EI approaches (e.g., Petrides and Furnham, 2000; Fernández-Berrocal and Extremera, 2006; Boyatzis et al., 2015) in a shared call for research that promotes a comprehensive vision of EI, one that acknowledges the unassailable contribution each existing measure, be they ability, self-report or behavioral EI, makes to the advancement of our understanding of what an emotional intelligent person thinks like, feels like and acts like.

## Author contributions

MT: responsible for the research design and writing of the first draft. XF-i-M: co-responsible for the data analysis. JB-F: co-responsible for the data analysis. RB: main revisions and comments. RS: responsible for the data collection, and secondary revisions and comments.

### Conflict of interest statement

The authors declare that the research was conducted in the absence of any commercial or financial relationships that could be construed as a potential conflict of interest.

## Footnotes

^1^By social courses we mean courses that have a substantial amount of social tasks, whereas analytic courses have a greater amount of analytic tasks.

^2^We borrow this term from economics and game theory, wherein two investment decisions are called *strategic complements* if they mutually reinforce one another, i.e., if an increase in the investment on one item raises the marginal return of the other. Otherwise when investment decisions offset one another they are called *strategic substitutes* (Bulow et al., 1985).

^3^See footnote 2.

^4^Specifically, in the Bayesian approach a parameter is assigned a prior distribution (based on previous research in the field), which is then updated with the observed data by means of a specified likelihood function, so as to produce a posterior distribution of the parameter (Wagner and Gill, 2005). In fact, the Bayesian approach is not entitled to use a p-value (as in frequentist statistics), as the probability of obtaining the observed data under the null hypothesis. Rather this approach yields information about the probability of the hypothesis given the data. As mentioned, the data is not a sample of a larger population but it is the population of all part-time and executive MBAs between 2006 and 2013 at an European business school.
